# 1 nm‐Resolution Sorting of Sub‐10 nm Nanoparticles Using a Dielectric Metasurface with Toroidal Responses

**DOI:** 10.1002/smsc.202300100

**Published:** 2023-08-17

**Authors:** Hong Luo, Xiang Fang, Chengfeng Li, Xinhua Dai, Ning Ru, Minmin You, Tao He, Pin Chieh Wu, Zhanshan Wang, Yuzhi Shi, Xinbin Cheng

**Affiliations:** ^1^ Institute of Precision Optical Engineering School of Physics Science and Engineering Tongji University Shanghai 200092 China; ^2^ MOE Key Laboratory of Advanced Micro-Structured Materials Shanghai 200092 China; ^3^ Shanghai Institute of Intelligent Science and Technology Tongji University Shanghai 200092 China; ^4^ Shanghai Frontiers Science Center of Digital Optics Shanghai 200092 China; ^5^ Technology Innovation Center of Mass Spectrometry for State Market Regulation Center for Advanced Measurement Science National Institute of Metrology Beijing 100029 China; ^6^ National Key Laboratory of Advanced Micro and Nano Manufacture Technology Shanghai Jiao Tong University Shanghai 200240 China; ^7^ Department of Photonics National Cheng Kung University Tainan 70101 Taiwan; ^8^ Center for Quantum Frontiers of Research & Technology (QFort) National Cheng Kung University Tainan 70101 Taiwan

**Keywords:** 1 nm resolution, dielectric metasurface, optofluidic sorting, sub-10 nm nanoparticles, toroidal dipole

## Abstract

Sorting nanoparticles is of paramount importance in numerous physical, chemical, and biomedical applications. Current technologies for sorting dielectric nanoparticles have a common size limit and resolution approximately of 20 and 10 nm, respectively. It remains a grand challenge to push the limit. Herein, the new physics that deploys toroidal and multipole responses in a dielectric metasurface to exert strong and distinguishable optical forces on sub‐10 nm nanoparticles is unravelled. The electric toroidal dipole, electric dipole, and quadrupole emerge with distinct light and force patterns, which can be leveraged to promise unprecedented high‐precision manipulations, such as sorting sub‐10 nm polystyrene nanoparticles at 1 nm resolution, sorting 20 nm proteins/exsomes at 3 nm resolution, conveying, and concentrating 100 nm gold nanoparticles. Remarkably, the design can also be employed to screen out medium‐sized nanoparticles from a mixture of nanoparticles with over three sizes. This optofluidic manipulation platform opens the new way to explore intriguing optical modes for the powerful manipulation of nanoparticles with nanometer precisions and low laser powers.

## Graphical abstract

1 nm‐precision sorting of sub‐10 nm polystyrene nanoparticles and 3 nm‐precision sorting of exsomes can be achieved by exploiting the electromagnetic toroidal dipole or multipoles in a dielectric metasurface. Medium‐sized nanoparticles can be screened out from a mixture of nanoparticles. This optofluidic platform opens the new way to explore intriguing optical modes for the nanometer‐precision manipulation with low laser powers.

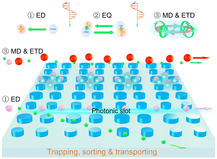

## 1 Introduction

Nanoparticles have been widely used in diverse areas such as physics, biology, medicine, catalysis, and cosmetics, where narrowing the size range becomes a key issue in quality control.^[^ ^1^, ^2^, ^3^ ^]^ For instance, optical properties (e.g., plasmon resonance and Raman spectra) of nanoparticles correlate tightly with the particle size, playing important roles in the biosensing and thermal therapy. The particle size can also significantly influence the turnover frequency in heterogeneous catalysis.^[^ ^4^, ^5^ ^]^ Thus, sorting of nanoparticles is becoming progressively crucial.

A great diversity of contemporary methodologies has been deployed for sorting nanoparticles such as microfluidics, acoustic tweezers, dielectrophoresis, magnetic beads, optical tweezers, etc. Most of them are difficult to handle sub‐100 nm dielectric nanoparticles.^[^ ^6^ ^]^ The flow cytometry confines particles in a hydrodynamic focusing, and sorts them with external force fields such as the electric field.^[^ ^7^, ^8^ ^]^ This method is well known as an efficient tool for manipulating cells, while needs fluorescence labeling (called fluorescence‐activated cell sorting) for smaller bioparticles, e.g., bacteria.^[^ ^8^, ^9^ ^]^ Acoustic tweezers can isolate small exsomes from blood samples, but are inefficient to discriminate two kinds of exsomes with similar sizes.^[^ ^10^ ^]^ By labeling bioparticles with magnetic beads, it enables a fast and high‐throughput screening, while lacks the ability of label‐free sorting.^[^ ^11^ ^]^ Dielectrophoresis uses strong electric fields to sort particles, resulting in a relatively low spatial resolution in handling tiny bioparticles such as bacteria and viruses.^[^ ^12^ ^]^ Remarkably, a promising technique called deterministic lateral displacement emerges in recent years as a star in fractionating exsomes and colloids down to 20 nm.^[^ ^13^ ^]^ However, the nanogap between nanopillars could be stuck by bioparticles or impurities, and the screening resolution is greater than 50 nm, meaning that 20 nm exsomes are difficult to be separated from 60 nm ones.

Optical tweezers, a Nobel Prize‐winning technique,^[^ ^14^, ^15^, ^16^ ^]^ represent a precise tool to manipulate nanoparticles by leveraging optical forces that alter dramatically with the particle size. They have been extensively studied and demonstrated to be paradigms in sorting nanoparticles in the last two decades due to the enriched understanding of light–matter interactions.^[^ ^17^, ^18^ ^]^ Examples include the sorting of microparticles with different sizes or refractive indices using holographic optical tweezers^[^ ^19^, ^20^ ^]^ and interferences waves,^[^ ^21^ ^]^ optical chromatography,^[^ ^22^ ^]^ waveguides,^[^ ^23^ ^]^ and optical switch.^[^ ^24^ ^]^ For single nanoparticles including metallic ones, 100 and 130 nm gold nanoparticles can be separated bidirectionally using plasmon resonances in two counterpropagating evanescent waves.^[^ ^25^ ^]^ Using the impinging flow to create a stagnant region, 50 and 100 nm gold nanoparticles can travel to different outlets by the optical radiation pressure.^[^ ^26^ ^]^ According to different quantum mechanical properties, nanodiamonds (diameter ≈50 nm) with and without nitrogen vacancy centers can be separated.^[^ ^27^ ^]^ Besides, sub‐100 nm gold nanoparticles can be separately trapped in a loosely overdamped system using a quasi‐Bessel beam^[^ ^28^ ^]^ or exquisitely designed phase fields.^[^ ^29^, ^30^ ^]^ Using the combination of spin and radiation pressure forces, Valero et al. demonstrated the aggregation of 15–30 nm gold nanoparticles, and moving away larger ones (e.g., 50 nm) near a silicon nanocube illuminated with a circularly polarized light.^[^ ^31^ ^]^ Recently, Xu et al. utilized the optomechanical wagon‐wheel effect to sort sub‐200 nm polystyrene nanoparticles.^[^ ^32^ ^]^ For a large quantity of nanoparticles, hundreds of 200 and 300 nm nanoparticles,^[^ ^33^ ^]^ or bacteria with different shapes^[^ ^34^ ^]^ can be selectively trapped in nanowaveguide pair arrays. So far, the size and resolution limits for optical sorting of single dielectric nanoparticles are 50 and 10 nm, respectively, while sorting capabilities can be compromised for massive nanoparticles.

Here, we put forward a new strategy to exploit strong light confinements and unique field patterns from combined dipole and multipole modes in a dielectric metasurface. Compared with plasmonic counterparts, dielectric nanostructures greatly mitigate the heating effect, thus being friendly to bioparticles. Meanwhile, multipole and toroidal modes with distributions of looped electric currents and magnetic fluxes could result in strong resonances of electric and magnetic fields that significantly enhance optical forces.^[^ ^35^, ^36^ ^]^ Though the magnetic toroidal dipole has been mathematically demonstrated to have the capability in enhancing the optical force,^[^ ^37^ ^]^ how to leverage the toroidal dipole in a nanostructure for manipulating nanoparticles remains unexplored. In this work, we design a dielectric metasurface that consists of four silicon disks in each unit cell to excite electric dipole and quadrupole, as well as electric toroidal dipole^[^ ^36^, ^38^, ^39^, ^40^ ^]^ in the communication band. Intriguingly, those optical modes promise unprecedented manipulation capabilities which are able to sort sub‐10 nm dielectric nanoparticles at an ultrahigh resolution of 1 nm, pick out medium‐sized nanoparticles, and transport nanoparticles along a photonic slot.

## 2 Results and Discussion

### 2.1 Exploiting Intriguing Optical Modes in a Dielectric Metasurface for Ultrahigh Precision Manipulation of Nanoparticles

**Figure 1:**
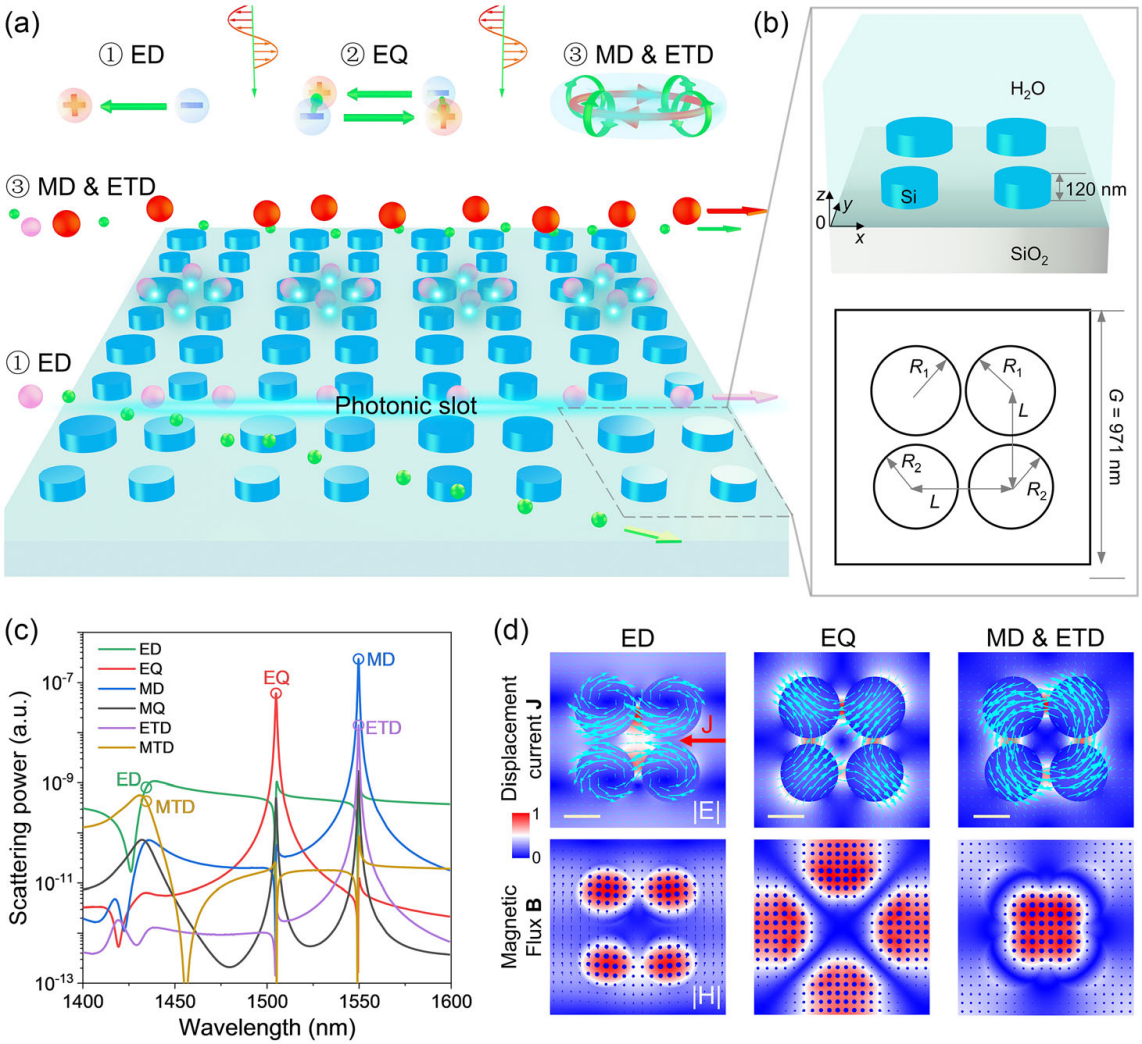
Electric and magnetic dipoles and multipoles for multifunctional and high‐precision manipulation of nanoparticles. a) Schematics of the ETD, ED, and EQ emerged by the illuminating a nanopillar array using a normally incident plane wave with the electric field along the *x* direction. With the ETD, medium‐sized nanoparticle can be trapped inside hotspots, while other nanoparticles flushed away by the flow stream. With the ED, nanoparticles can be sorted and pushed along a photonic slot. b) Structural parameters of a unit cell consisting of four silicon pillars on a silicon oxide substrate. The nanopillars have a height of 120 nm and are imbedded in water. Two nanopillars in the upper low have a radius *R*_1_ = 170 nm; the other two in the lower low have a radius *R*_2_ = 160 nm. The distance between two nanopillars *L* = 360 nm, and the length of each unit cell *G* = 971 nm. c) Multipole expansion. ED, electric dipole; MD, magnetic dipole; EQ, electric quadrupole; MQ, magnetic quadrupole; ETD, electric toroidal dipole; MTD, magnetic toroidal dipole. d) Analysis of different optical modes by plotting the displacement current and magnetic flux. The normalized electric and magnetic fields are plotted at *z* = 60 nm. Scale bars equal 200 nm.

The schematic of the multifunctional manipulation of nanoparticles using a dielectric metasurface is shown in **Figure** **1**a. The metasurface consists of nanopillar arrays on a silicon oxide substrate. Each unit cell is imbedded in water and composed of four silicon nanopillars with a height of 120 nm (Figure 1b). The four pillars have two radii to break the symmetry and induce strong electromagnetic resonances (Figure 1c). By sweeping the wavelength of light (*x* polarization and propagating along the *z* direction), three modes emerge, e.g., electric dipole (ED), electric quadrupole (EQ), and magnetic dipole (MD) & electric toroidal dipole (ETD), which are also manifested in the multipole expansion in Figure 1c and transmittance spectrum in Figure S1, Supporting Information. The scattering intensity of each electromagnetic multipole can be expressed as^[^ ^41^, ^42^ ^]^

(1) $$I_{\text{sca}}=\frac{2\omega^{4}}{3c^{3}}\left[{\left|\vec{p}\right|}^{2}+{\left|\vec{m}\right|}^{2}\right]+\frac{2\omega^{6}\varepsilon_{m}}{3c^{5}}\left[\varepsilon_{0}\mu_{0}\varepsilon_{m}{\left|{\vec{T}}^{\left(e\right)}\right|}^{2}+{\left|{\vec{T}}^{\left(m\right)}\right|}^{2}\right]+\frac{\omega^{6}}{5c^{5}}{\sum \left|Q_{\alpha \beta }^{\left(e\right)}\right|}^{2}+\frac{\omega^{6}}{40c^{5}}{\sum \left|Q_{\alpha \beta }^{\left(m\right)}\right|}^{2}$$

where *α*, *β* = *x*, *y*, *z*, *ε*_*m*_ is the permittivity of the medium, and $\vec{p}$, $\vec{m}$, ${\vec{T}}^{\left(e\right)}$, ${\vec{T}}^{\left(m\right)}$, $Q_{\alpha \beta }^{\left(e\right)}$, and $Q_{\alpha \beta }^{\left(m\right)}$ denote the ED, MD, ETD, magnetic toroidal dipole (MTD), EQ, and magnetic quadrupole in Cartesian coordinate, respectively.

Intriguingly, the three modes correlate with a set of light patterns that promise multifunctional and high‐precision manipulation of nanoparticles. The ED and EQ appear evidently from the electric moments in four disks with different radii. The ETD originates from the trapped mode in coupled and asymmetrically distributed nanopillars, which can be visualized from the closed loop of displacement currents that penetrates all four nanopillars and magnetic fluxes that are directed along the torus meridians, as shown in the right column of Figure 1d. It is worth noting that distributions of MD and ETD are very similar, and hereafter, we refer to this mode only to ETD because the MTD is commonly explored rather than the ETD.^[^ ^39^, ^43^, ^44^, ^45^ ^]^ Meanwhile, the ED mode in the left column of Figure 1d can be interpreted from displacement currents between upper and lower nanopillars. And the EQ mode is the dominant optical mode near 1500 nm in Figure 1c, which can be easily visualized from displacement currents and magnetic fluxes in the middle column of Figure 1d. Notably, optical modes here are different from those in the previous work^[^ ^36^ ^]^ because of different structural parameters. More simulation results about the design of multipoles can be found in Figure S2, Supporting Information. In the practical fabrication process, there exists the inevitable fabrication tolerance in the sample, resulting in the shift of the resonance peak or dip in the spectrum, as shown in Figure S2, Supporting Information. In this case, one should initially measure the spectrum and find the resonance wavelength for the effective manipulation. Therefore, a tunable laser is suggested to be used to excite the correct optical mode for the precise optical manipulation of nanoparticles because the laser power used in this work is sufficiently low (see simulation results in the following content).

### 2.2 Theory of Optical Forces

In the dipole theory, the optical force on a small nonmagnetoelectric dipole can be expressed as

(2) F=Fgrad+Frad+Fcurl=Re(αe)4∇|E|2+σextnmcP−σextc2nm∇×Se

where *α*_*e*_ is the electric polarizability,^[^ ^18^, ^46^, ^47^ ^]^ *n*_*m*_ is the refractive index of the embedding medium, *σ*_ext_ is the particle extinction cross section, **P** is the Poynting vector, and *c* is the light speed in vacuum. **S**_e_ is the spin angular momentum, which can expressed as S=Im(εE*×E)/4ω, where *ε* is the permittivity of the medium, and *ω* is the angular frequency of light. Normally, the force from the curl of the spin angular momentum (known as the Belinfante spin momentum) is smaller than those from the electric field gradient and Poynting vector, especially for linearly polarized incident light.^[^ ^46^, ^48^ ^]^ Thus, the optical force in this work can be further simplified as

(3) F=Fgrad+Frad=Fgrad+Fsca+Fabs≈2πnmr3c(m2−1m2+2)∇I +128π5nmr6I3cλ4(m2−1m2+2)2+8π2nmr3IcλIm(m2−1m2+2)

where *I* is the light intensity, $m=n_{1}/n_{m}$, with *n*_1_ being the refractive index of the particle.

We will not use Equation (2) and (3) for a rigorous calculation of optical force in this work, while it can be used to interpret particle dynamic behaviors in distinct light fields. Instead, for a rigorous calculation, Minkowski stress tensor is implemented in COMSOL, which can be given as^[^ ^49^ ^]^

(4) $$\left\langle F_{\text{OLF}}\right\rangle =\oint_{S}\left\langle \overset{↔}{T}\right\rangle \cdot \hat{n}dA$$

(5) $$\left\langle T_{ij}\right\rangle =\frac{1}{2}\left[D_{i}E_{j}^{*}+B_{i}H_{j}^{*}-\frac{1}{2}\left(D\cdot E^{*}+B\cdot H^{*}\right)\delta_{ij}\right]$$

where $\hat{n}$ is the unit outward normal to the integral surface, and $\delta_{ij}$ is the Kronecker delta. The obtained optical force is then used to calculate the potential energy *U* from the integral of force in two dimensions.^[^ ^33^ ^]^

### 2.3 Nanometer‐Precision Sorting of Nanoparticles Using the Electric Toroidal Mode

**Figure 2:**
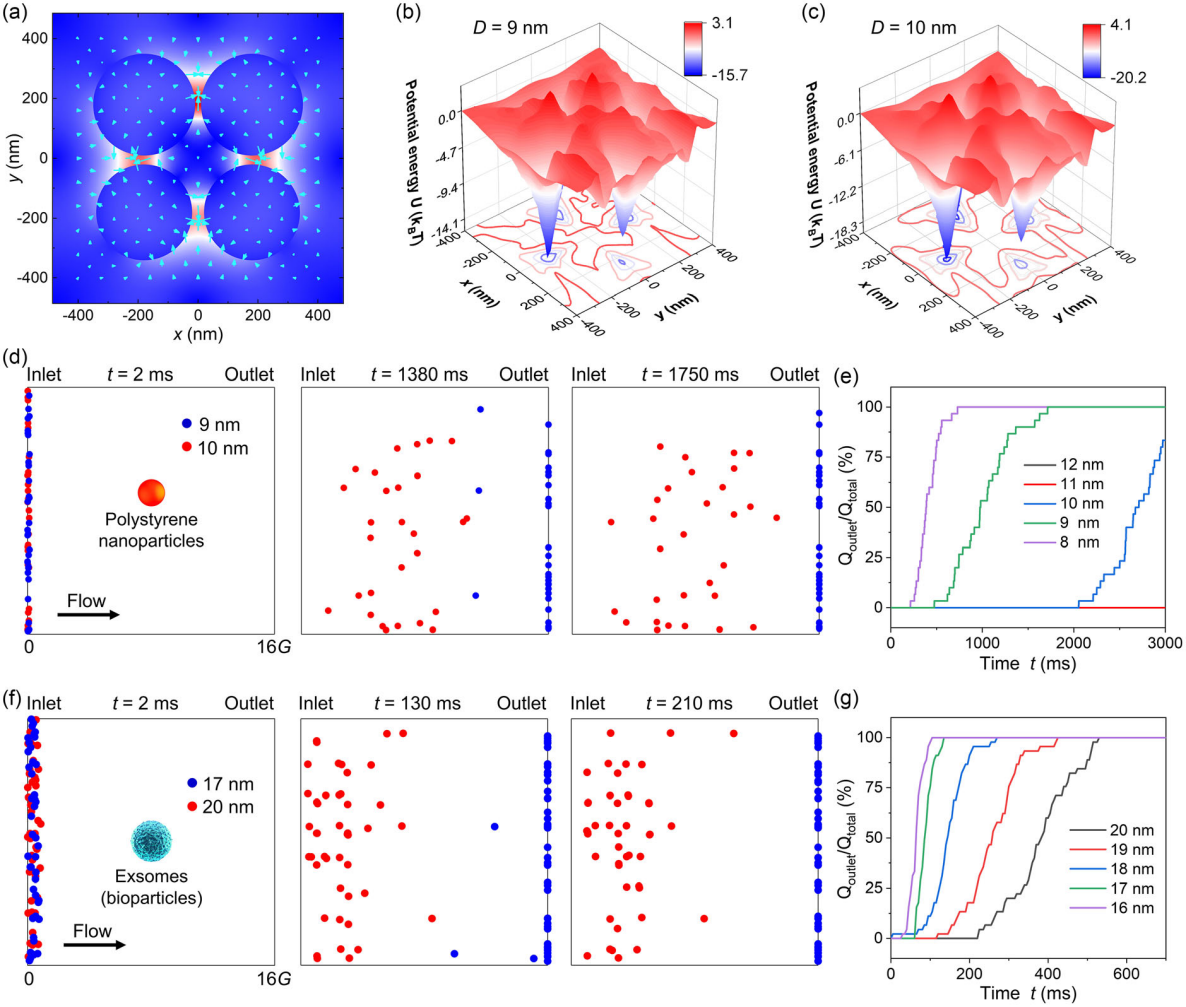
High‐resolution sorting of polystyrene nanoparticles and exsomes at the wavelength of 1550 nm. a) Plot of the electric field and force vectors on a 10 nm polystyrene (RI = 1.58) nanoparticle for the ETD mode. The four electric hotspots exert optical gradient forces on the nanoparticle. b,c) Potential wells for 9 nm (b) and 10 nm (c) polystyrene nanoparticles. *I* = 10 mW μm^−2^ in (b) and (c). d) Simulated video frames of sorting of 9 and 10 nm polystyrene nanoparticles. All 10 nm nanoparticles reach the outlet on the right side at *t* = 1.75 s, while no 9 nm nanoparticles reach the outlet at that time. The separation of the two kinds of nanoparticles can be prominent when we enlarge the working range to, for example, 100 μm. e) Quantified percentage of 8–12 nm polystyrene nanoparticles to the outlet with time. f) Simulated video frames of sorting of 17 and 20 nm exsomes. All 17 nm nanoparticles reach the outlet on the right side at *t* = 210 s, while no 20 nm nanoparticles reach the outlet at that time. g) Quantified percentage of 16–20 nm exsomes to the outlet with time. In (d) and (e), *I* = 19 mW μm^−2^, *v* = 80 μm s^−1^. In (f) and (g), *I* = 9.5 mW μm^−2^, *v* = 300 μm s^−1^.

The electric field exhibits four focused hotspots in the ETD, as shown in **Figure** **2**a. Those hotspots exert strong optical gradient forces on 10 nm polystyrene nanoparticles to attract them inside, as elucidated from force arrows in Figure 2a. As potential well depths of 8–12 nm polystyrene nanoparticles increase linearly with their sizes (Figure 2b,c and S3, Supporting Information), we can selectively trap target large nanoparticles and release small ones. For example, 10 nm nanoparticles can be trapped inside potential wells, while all 9 nm nanoparticles flow into the outlet on the right size in 1.75 s (Figure 2d), achieving a remarkable sorting resolution of 1 nm. Trajectories of 9 and 10 nm nanoparticles experiencing the optical force, fluidic drag force, and Brownian force are simulated in COMSOL, as shown in Figure 2e and Movie S1, Supporting Information, demonstrating the high‐efficiency and high‐resolution sorting of both particles when the laser intensity *I* = 19 mW μm^−2^ and the flow velocity *v* = 80 μm s^−1^. The separation completes at 1.75 s when all 9 nm nanoparticles flow into the outlet, while 10 nm nanoparticles are retained in potential wells and have not yet reached the right side. Currently, the simulation region in Figure 2e is only 16* G* (≈15.5 μm). The separation result can be much more prominent when extending the trapping region to, for example, 100 μm. Notably, the screening capability for bioparticles (e.g., exsomes/proteins, refractive index ≈1.4) retards due to the weaker optical force from the lower refractive index. The 20 nm exsomes can be separated from 17 nm entities when the laser intensity is 9.5 mW μm^−2^ and the flow velocity is 300 μm s^−1^, as shown in Figure 2f,g. More simulation results can be found in Figure S3–S5, Supporting Information. The separation velocity for 10 nm and 11 nm polystyrene nanoparticles can reach 200 μm s^−1^ at *I* = 13.8 mW μm^−2^, as shown in Figure S3, Supporting Information.

### 2.4 Screening out Medium‐Sized Nanoparticles from a Bunch of Nanoparticles

**Figure 3:**
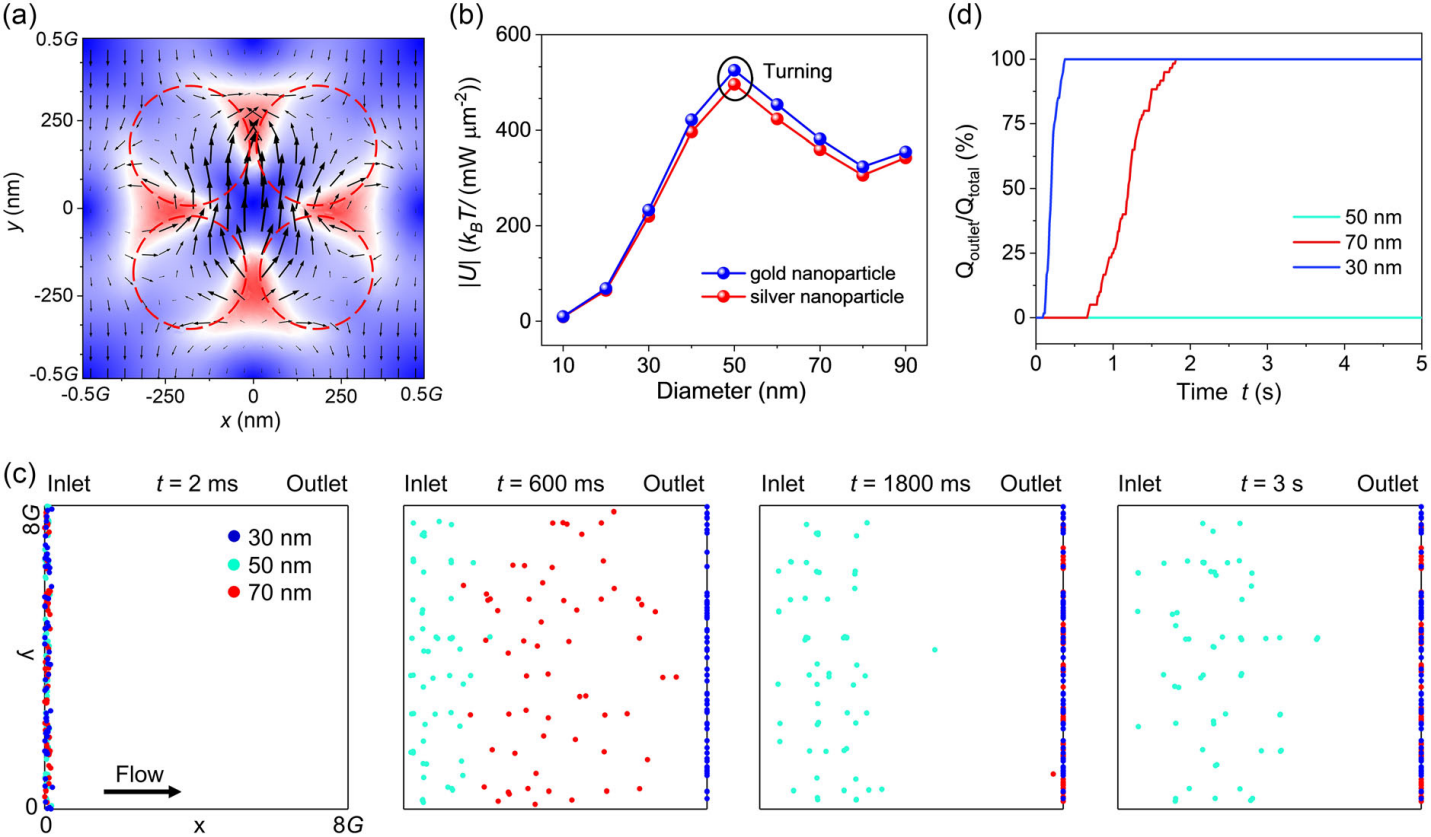
Screening medium‐sized nanoparticles from a bunch of nanoparticles using an ultralow laser intensity (0.036 mW μm^−2^) at the wavelength of 1550 nm. a) Plot of the electric field and Poynting vector of the ETD mode at *z* = 150 nm. The four electric hotspots tend to trap nanoparticles inside by the optical gradient force, while Poynting vectors tend to push away nanoparticles by the radiation pressure. The potential well depth thus comes from the competition of two forces. The electric field and Poynting vectors are plotted at a plane 30 nm above the substrate. b) Potential well depth |*U*| versus the diameter of nanoparticle. In the range of 10–90 nm, for both gold and silver nanoparticles, |*U*| initially increases and then drops with the particle size at a turning point of 50 nm due to the large radiation pressure on big nanoparticles. Thus, we can only trap 50 nm nanoparticles and release 30 and 70 nm nanoparticles by controlling the laser power and flow velocity. c) Video frames of the screening 50 nm polystyrene nanoparticles from 30 and 70 nm ones. 30 nm (blue) and 70 nm (red) nanoparticles successively move to the outlet on the right side by the fluidic drag force. In contrast, 50 nm nanoparticles are trapped inside hotspots in the flow stream. *I* = 0.036 mW μm^−2^, and *v* = 50 μm s^−1^. d) Quantified percentage of nanoparticles to the outlet with time. 30 and 70 nm nanoparticles move to the outlet in 0.6 and 1.8 s, respectively.

Counterintuitively, the ETD mode can also be utilized to screen out the medium‐sized nanoparticle from a mixture of different‐sized nanoparticles. This is due to the combined effect of the electric field and energy flow (Poynting vector), as shown in **Figure** **3**a. The focused electric field exerts a strong optical gradient force on nanoparticles (see Figure 2). This optical gradient force becomes dominant and surpasses the radiation pressure force when the particle size is sufficiently small since $F_{\text{grad}}\propto r^{3}$ and $F_{\text{sca}}\propto r^{6}$, as estimated by Equation (3). The optical scattering force can be greater than the optical gradient force when the particle size increases. Consequently, the potential well unintuitively can be shallower for larger nanoparticles, for instance, 50 versus 70 nm in Figure 3b. When the particle size becomes even greater, the dipole approximation does not hold, and the back action effect^[^ ^50^, ^51^ ^]^ from the particle interfering the light field becomes prominent; thus the depth of potential well arises again. The 50 nm gold or silver nanoparticle with a deeper potential well can be trapped, while 30 and 70 nm nanoparticles with shallower potential wells are released, using an ultralow laser intensity of 0.036 mW μm^−2^ and a flow velocity of 50 μm s^−1^. 30 nm gold nanoparticles arrive earlier to the outlet than 70 nm gold nanoparticles due to the shallower potential well of the 30 nm nanoparticle, as shown in Figure 3c and Movie S2, Supporting Information. 50 nm gold nanoparticles are trapped in hotspots arising from the toroidal dipole. Percentages of nanoparticles moving to the outlet are quantified in Figure 3d. It takes ≈700 ms for the first and near 1.8 s for the last 70 nm gold nanoparticles to reach the outlet on the right, respectively. Using the same strategy, we can screen out 60 nm nanoparticles from a mixture of 30, 60, and 70 nm nanoparticles.

### 2.5 Fast Sorting and Transporting of Nanoparticles Using a Photonic Slot

**Figure 4:**
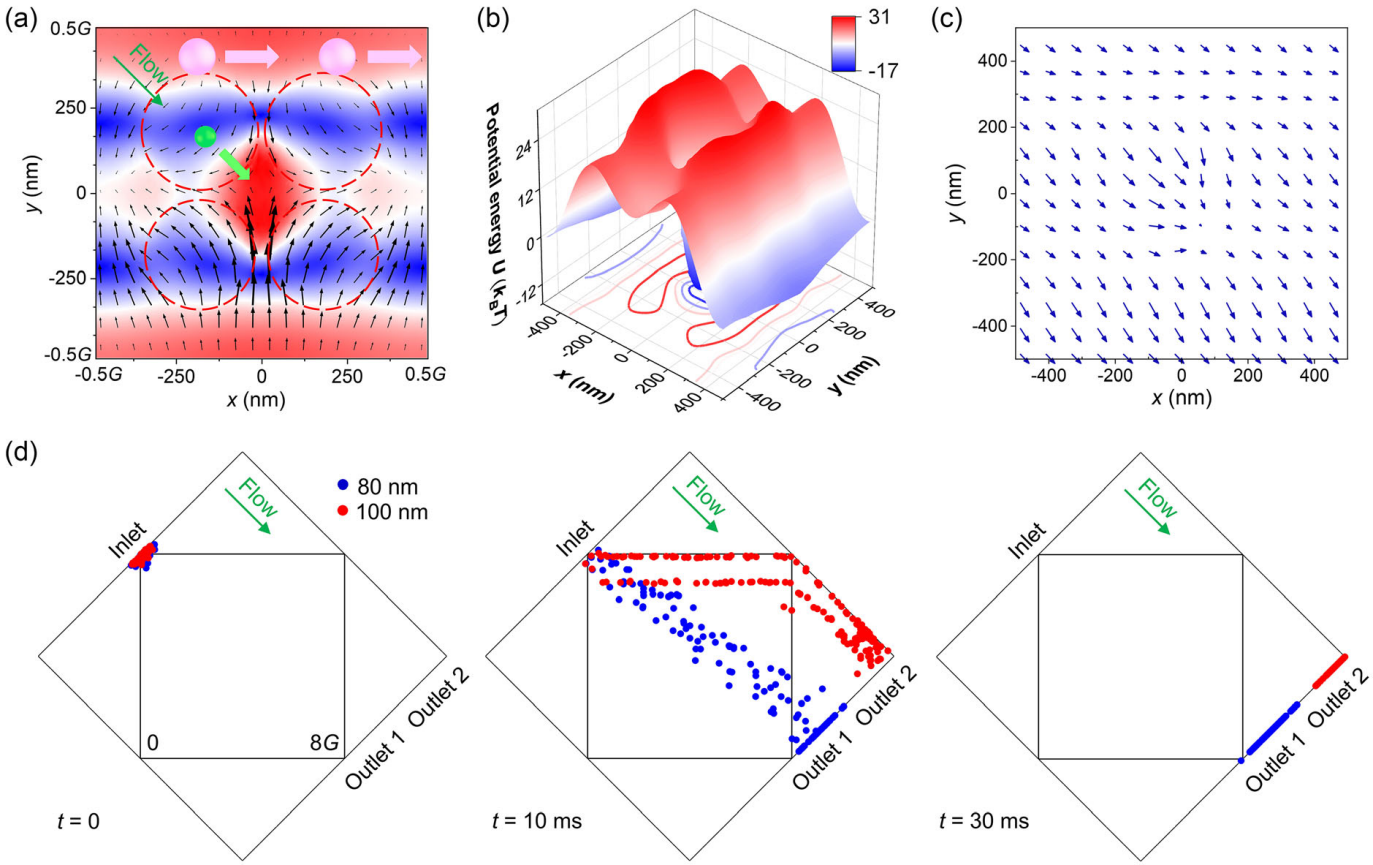
Fast sorting and transporting of gold nanoparticles by the photonic slot in the electric dipole mode at the wavelength of 1435.6 nm. a) Plot of the electric field and Poynting vector of the ED mode at *z* = 150 nm. When implementing a tilted flow stream, the small nanoparticle (green) is carried away by the flow stream, while the large nanoparticle (pink) is confined in the photonic slot by the optical gradient force and transported by the fluidic drag force in the *x* direction. b) Potential well of the 80 nm gold nanoparticle. A photonic slot and a hole occur in the edge and in the middle of a unit cell, respectively. c) Plot of the total force (optical force + fluidic drag force) on an 80 nm gold nanoparticle. d) Video frames of sorting and transporting gold nanoparticles. 80 nm gold nanoparticles (blue) move along the fluidic drag force, while 100 nm gold nanoparticles (red) are confined in photonic slot and transported by the fluidic drag force in the *x* direction. In (b−d), *I* = 2.1 mW μm^−2^, and *v* = 1500 μm s^−1^.

The ED mode not only has two hotspots surrounded by four nanopillars, but also has a slot between adjacent up and down unit cells at *z* = 60 nm (half the height of the nanopillar, see Figure 1b,d). The two hotspots can merge into one, and the slot becomes prominent above the nanopillar (at *z* = 150 nm), as shown in **Figure** **4**a. When implementing a tilted fluid, the small nanoparticle (green) that influences smaller optical force moves with the fluid stream. The large nanoparticle (pink) that experiences larger optical gradient force from the slot in the *y* direction will move along the slot in the *x* direction, thus can be separated from the small one. The potential well profile of the 80 nm gold nanoparticle in Figure 4b shows a slot and a valley with similar depths, thus the nanoparticle tends to be trapped inside the hotspot or the slot. It is worth noting that the 80 nm gold nanoparticle detaches from the trapping hotspot and moves toward the direction of fluid when applying a fluidic drag force that devastates the potential well, as shown in Figure 4c,d. The 100 nm gold nanoparticle which experiences larger optical forces can easily hop from the single hotspot to the adjacent lower slot when applying a tilted fluidic drag force.^[^ ^52^, ^53^, ^54^ ^]^ It will then move along the photonic slot by the fluidic drag force in the *x* direction, as can be seen from trajectories of 100 nm gold nanoparticles in Figure 4d and Movie S3, Supporting Information. More simulation results of manipulating gold nanoparticles can be found in Figure S6, and S7, Supporting Information. As the gold nanoparticle normally experiences larger optical forces than the dielectric one, the fluid speed can reach 1500 μm s^−1^, which is considerably high in optical manipulation.

## 3 Discussion and Conclusion

Intriguing optical modes in a dielectric metasurface have previously shown great advantages in enhancing the electromagnetic field and optical forces on chiral^[^ ^35^ ^]^ and achiral^[^ ^42^, ^55^, ^56^, ^57^ ^]^ particles. Particularly, the ETD in this work shows an over 200‐fold enhancement in the electric field, outperforming previously reported nanocavities (≈70‐fold enhancement).^[^ ^58^ ^]^ Meanwhile, the ETD confines the electric field at a few tens of nanometers, providing an ideal paradigm to generate strong optical gradient forces for the selective trapping using a low laser intensity (as low as 0.036 mW μm^−2^). By combing the optical force with the fluidic drag force, 10 nm polystyrene nanoparticles can be trapped while 9 nm ones are released, manifesting a sorting resolution of 1 nm. For the capability in handling bioparticles, 20 nm exsomes can be screened out from 17 nm ones. Intriguingly, by leveraging the influence of optical radiation pressure to the potential well created by the optical gradient force, medium‐sized nanoparticles can be screened out from a mix of nanoparticles with three or more sizes. Utilizing the slot pattern from the ED, large nanoparticles can be confined and transported in the photonic slot, subsequently be screened out from small nanoparticles that move with the fluid.

Due to the negligible absorption of silicon and silicon oxide in the communication band, as well as the small input laser power/intensity, this optofluidic manipulation platform should cause a negligible temperature increasement, thus being friendly to bioparticles. This endows a certain advantage over plasmon nano‐optical tweezers^[^ ^59^, ^60^, ^61^, ^62^, ^63^ ^]^ or opto‐thermoelectric tweezers^[^ ^64^, ^65^, ^66^, ^67^, ^68^, ^69^ ^]^ because an increment of temperature in several degrees could do a great harm to bioparticles.^[^ ^70^, ^71^, ^72^, ^73^, ^74^ ^]^ Optical tweezers with all‐dielectric metastructures have promised a new realm in the low‐power, multifunctional, and tunable manipulation of polymers and bioparticles.^[^ ^18^, ^75^, ^76^, ^77^ ^]^

This work not only showcases the intriguing and effective example of optical manipulation using the dipole, multipole, and toroidal dipole in a dielectric metamaterial nanostructure, but also opens up a new gate for nanometer‐precision sorting and transporting of nanoparticles. It breaks the technical bottleneck and extends the current size limit and resolution in particle sorting realm to 10 and 1 nm, respectively. Thus, our work has great potential in exploiting intriguing optical modes in dielectric metasurfaces for biological applications,^[^ ^78^ ^]^ such as screening and diagnosis of bioparticles down to the sub‐10 nm scale.

## Conflict of Interest

The authors declare no conflict of interest.

## Author Contributions

Y.S. and X.C. conceived the idea. H.L., C.L., P.C.W., and Y.S. performed numerical simulations. All authors were involved in the discussion and analysis. Y.S., H.L., T.H., Z.W., and X.C. prepared the manuscript. X.F., Y.S., and X.C. coordinated all the work. All authors commented on the manuscript.

## Supporting information

Supplementary Material

## Data Availability

The data that support the findings of this study are available from the corresponding author upon reasonable request.
