# Supplementary material for: 1 nm‐Resolution Sorting of Sub‐10 nm Nanoparticles Using a Dielectric Metasurface with Toroidal Responses
Source: Small Sci. 2023 Aug 17;3(9):2300100. doi: 10.1002/smsc.202300100 (PMC11935857; doi:10.1002/smsc.202300100)
Supplement: Supplementary file 1 — Supplementary Material [file SMSC-3-2300100-s001.zip › smsc202300100-sup-0001-SuppData-S1/smsc202300100-sup-0001-SuppData-S1.pdf]

Supporting Information for

1-nm-resolution sorting of sub-10-nm nanoparticles using a dielectric metasurface with toroidal responses

Hong Luo[1,2,3,4], Xiang Fang[5*], Chengfeng Li[1,2,3,4], Xinhua Dai[5], Ning Ru[5], Minmin You[6], Tao He[1,2,3,4], Pin Chieh Wu[7], Zhanshan Wang[1,2,3,4], Yuzhi Shi[1,2,3,4]* and Xinbin Cheng[1,2,3,4]*

[1] Institute of Precision Optical Engineering, School of Physics Science and Engineering, Tongji University, Shanghai 200092, China

[2] MOE Key Laboratory of Advanced Micro-Structured Materials, Shanghai 200092, China.

[3] Shanghai Institute of Intelligent Science and Technology, Tongji University, Shanghai 200092, China

[4] Shanghai Frontiers Science Center of Digital Optics, Shanghai 200092, China

[5] Technology Innovation Center of Mass Spectrometry for State Market Regulation, Center for Advanced Measurement Science, National Institute of Metrology, Beijing 100029, China

[6] National Key Laboratory of Advanced Micro and Nano Manufacture Technology， Shanghai Jiao Tong University, Shanghai, 200240, China

[7] Department of Photonics, National Cheng Kung University, Tainan 70101, Taiwan

*Corresponding authors: fangxiang@nim.ac.cn (X.F.); yzshi@tongji.edu.cn (Y.S.); chengxb@tongji.edu.cn (X.C.)

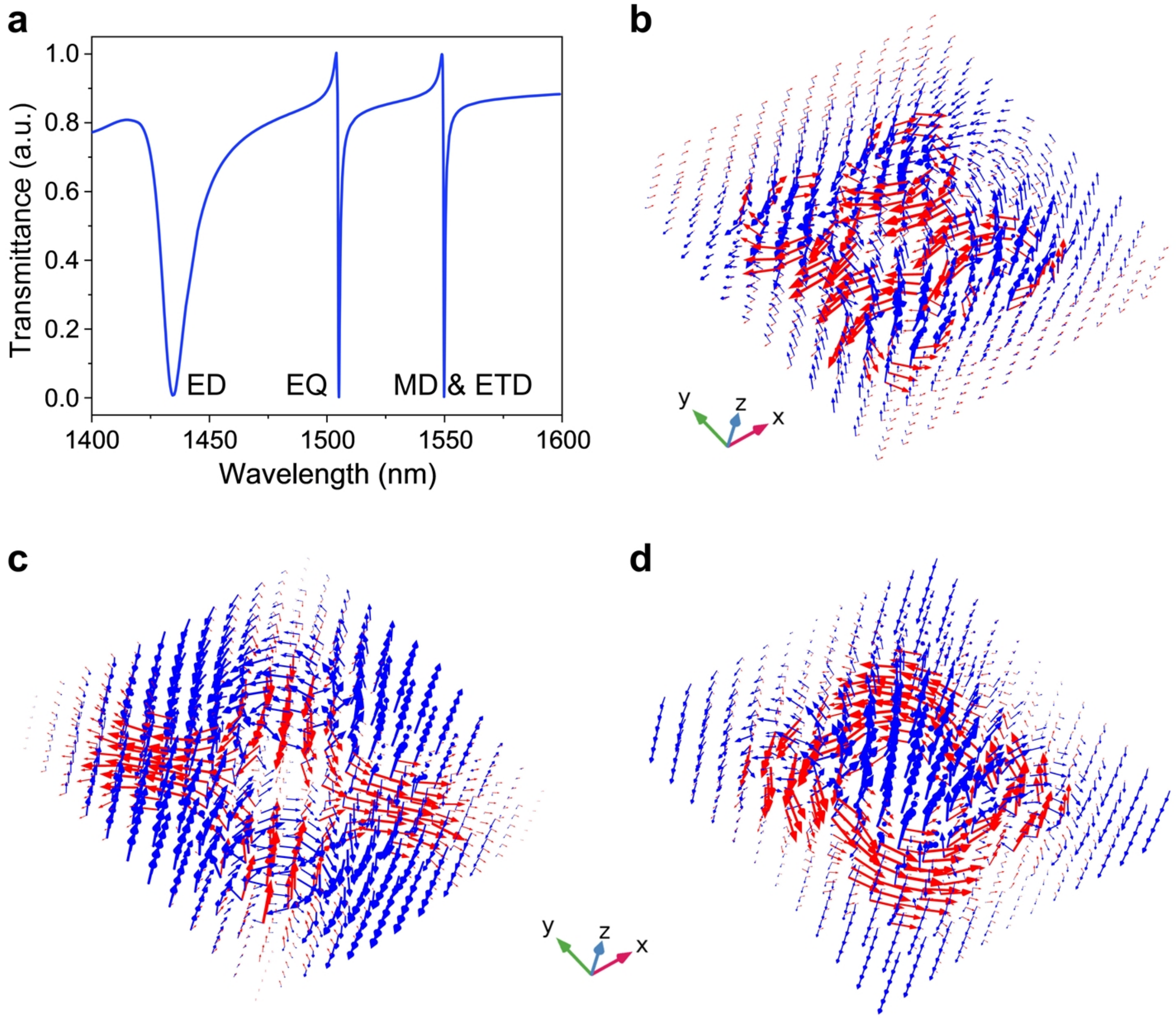

Fig. S1 | Analysis of optical modes in the dielectric metasurface (corresponding to Figs. 1c and 1d). (**a**) Transmittance spectrum. Three dips occur in the transmittance spectrum, representing the electric dipole (ED), electric quadrupole (EQ) and magnetic dipole (MD) & electric toroidal dipole (ETD). Plots of the electric displacement currents and magnetic fluxes of (**b**) ED, (**c**) EQ and (**d**) MD & ETD. In (**b**)−(**d**), blue and red arrows denote the magnetic flux and electric displacement current, respectively.

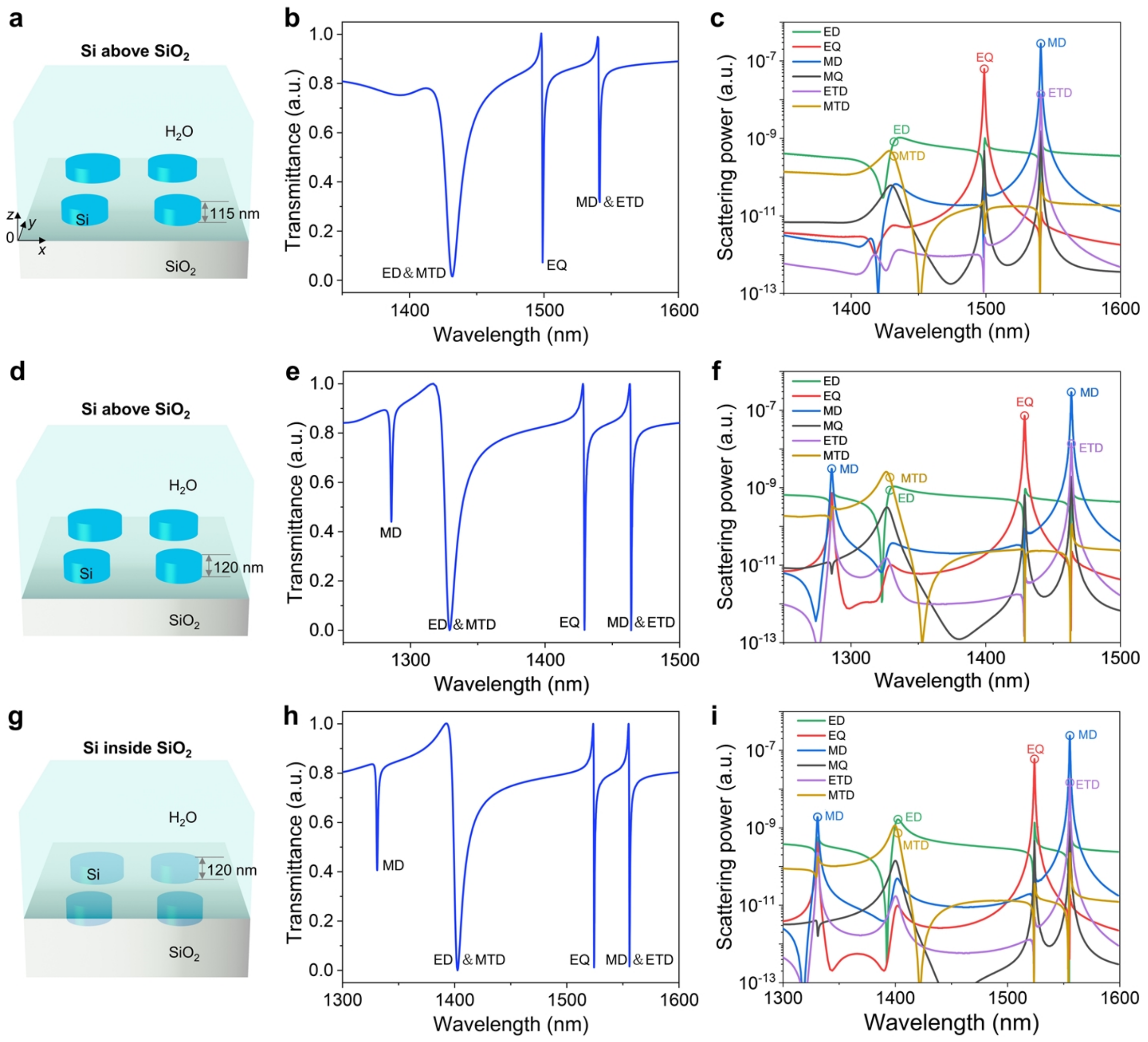

Fig. S2 | Design of multipoles in the dielectric metasurface. Silicon nanopillars with height (**a**)−(**c**) 115 nm and (**d**)−(**f**) 120 nm are placed above the silicon oxide substrate. (**h**)-(**i**) Silicon nanopillars are imbedded the silicon oxide substrate. (**b**), (**e**) & (**h**) Transmittance spectrum. (**c**), (**f**) & (**i**) Scattering power for the multipole expansion. ED: electric dipole; MD: magnetic dipole; EQ, electric quadrupole; MQ, magnetic quadrupole; ETD: electric toroidal dipole; MTD: magnetic toroidal dipole. In (**a**)−(**c**), R_1 = 170 nm, R_2 = 160 nm, L = 360 nm, G = 971 nm; In (**d**)−(**f**), R_1 = 170 nm, R_2 = 160 nm, L = 360 nm, G = 871 nm; In (**g**)−(**i**), R_1 = 180 nm, R_2 = 170 nm, L = 380 nm, G = 900 nm. The structural parameters except the height in (**a**)−(**c**) are the same as those in Fig. 1. The spectrum has a blue shift when the height is reduced.

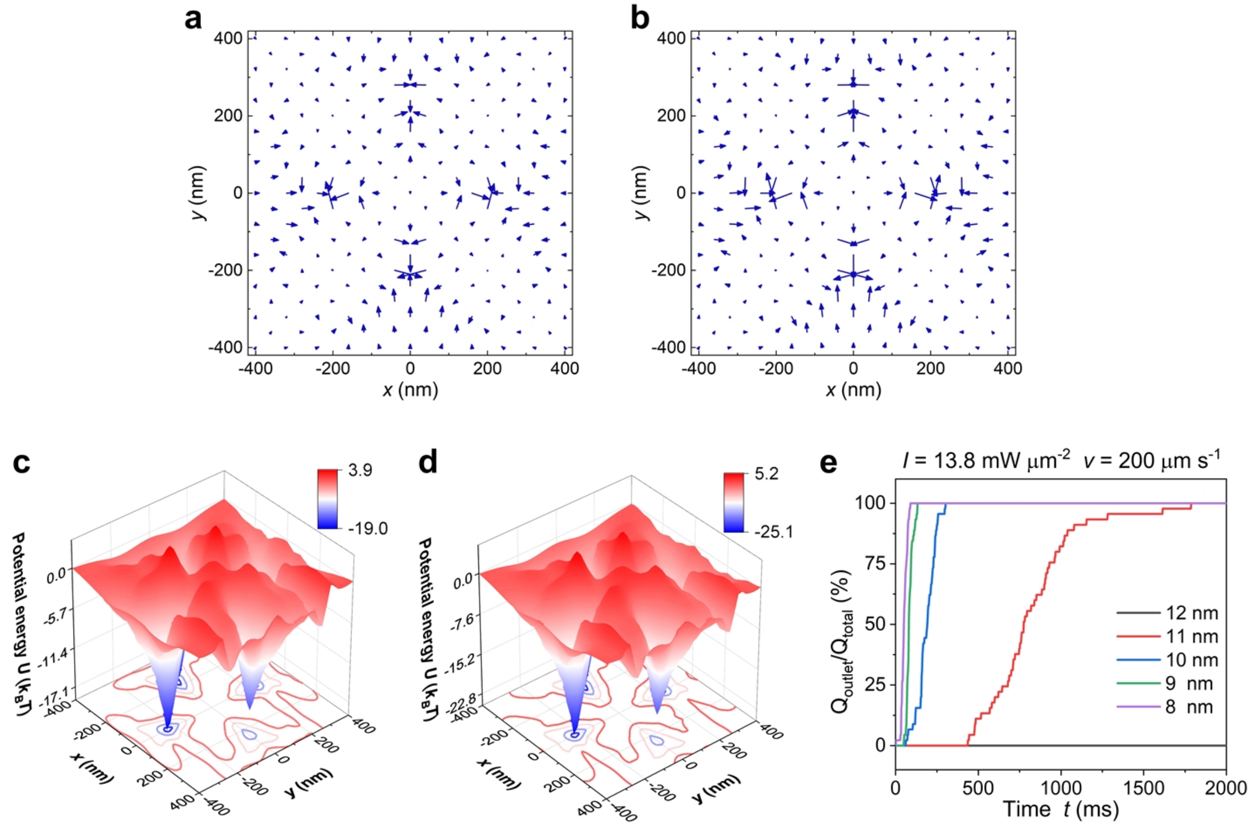

Fig. S3 | Simulations of optical forces and potential wells for the sorting of polystyrene nanoparticles in the ETD. Distributions of optical forces on (**a**) 10-nm and (**b**) 11-nm polystyrene nanoparticles. Potential wells of (**c**) 10-nm and (**d**) 11-nm polystyrene nanoparticles. **e**, Sorting mechanism by analysing the percentage of 8−12-nm nanoparticles reaching the outlet. Nanoparticles reaching the outlet can be separated from those trapped inside potential wells. In (**a**)−(**e**), I = 13.8 mW/μm^2. In (e), v = 200 μm/s.

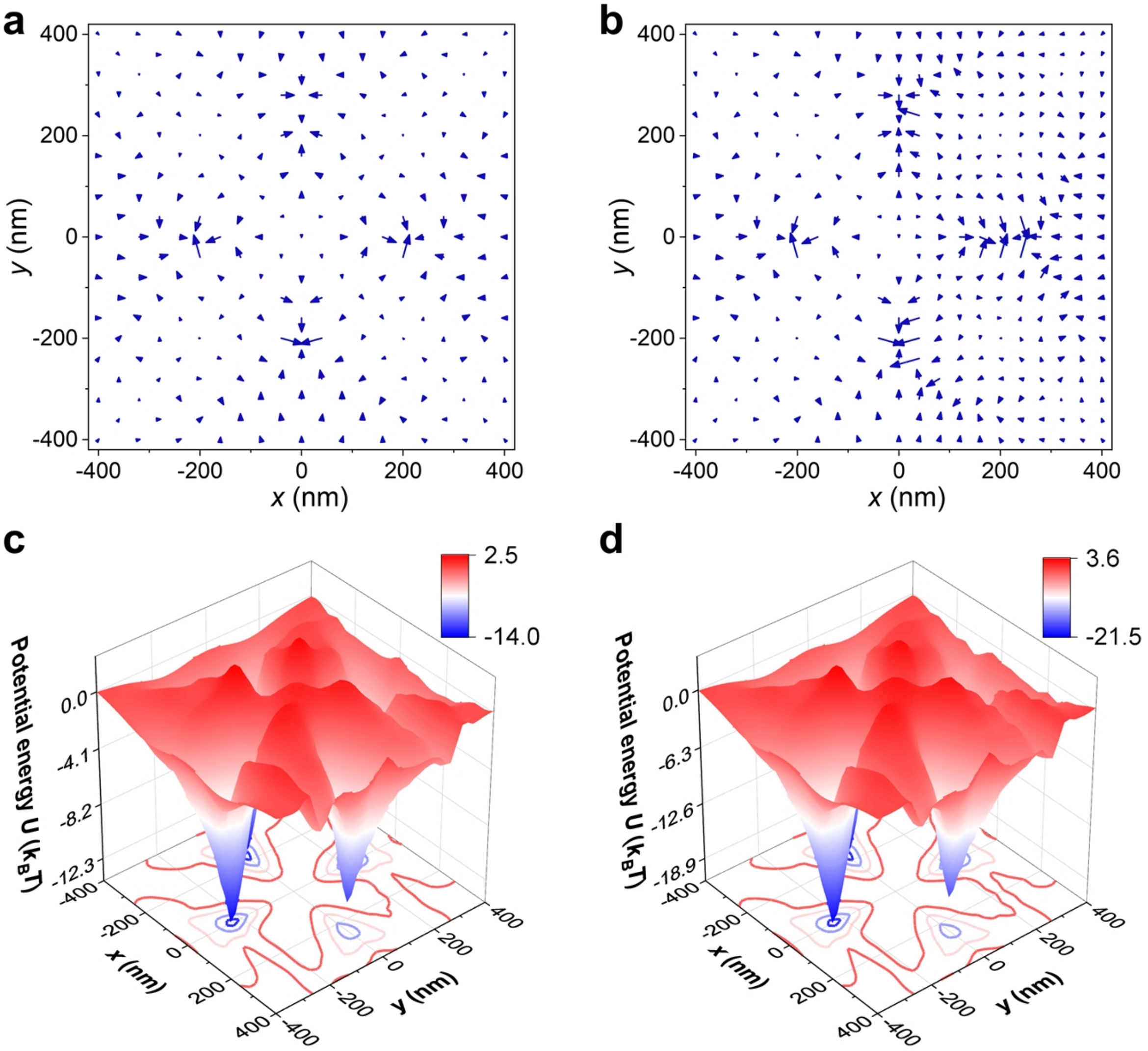

Fig. S4 | Simulations of optical forces and potential wells on exsomes in the ETD. Distributions of optical forces on (**a**) 10-nm and (**b**) 20-nm exsomes (RI = 1.4). Potential wells of (**c**) 10-nm and (**d**) 20-nm exsomes. In (**a**)−(**d**), $I = 9.5$ mW/μm^2, which is the same as Figs. 2f and 2e.

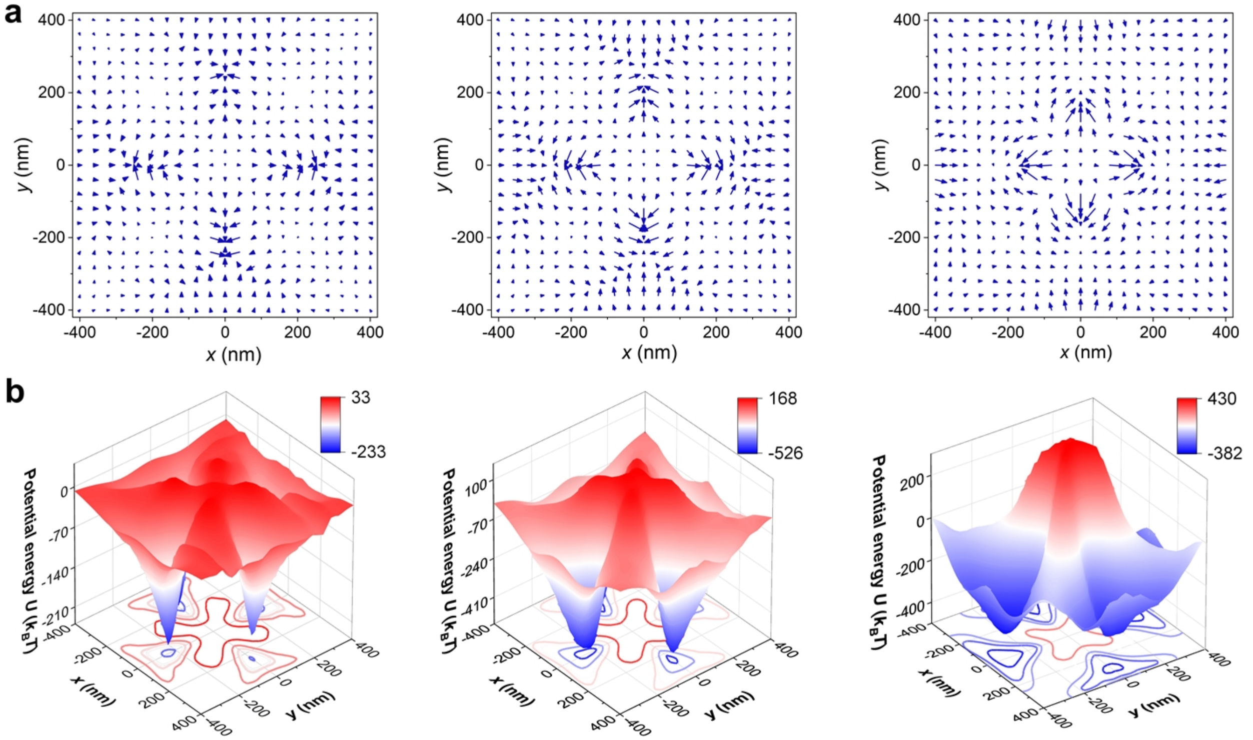

Fig. S5 | Simulations of optical forces and potential wells on gold nanoparticles in the ETD. (**a**) Distributions of optical forces on 30-nm, 50-nm and 70-nm gold nanoparticles. Potential wells of (**b**) 30-nm, 50-nm and 70-nm gold nanoparticles. The four hotspots in the ETD trap gold nanoparticles tightly in potential wells. In (**a**) and (**b**), $I = 1$ mW/μm^2.

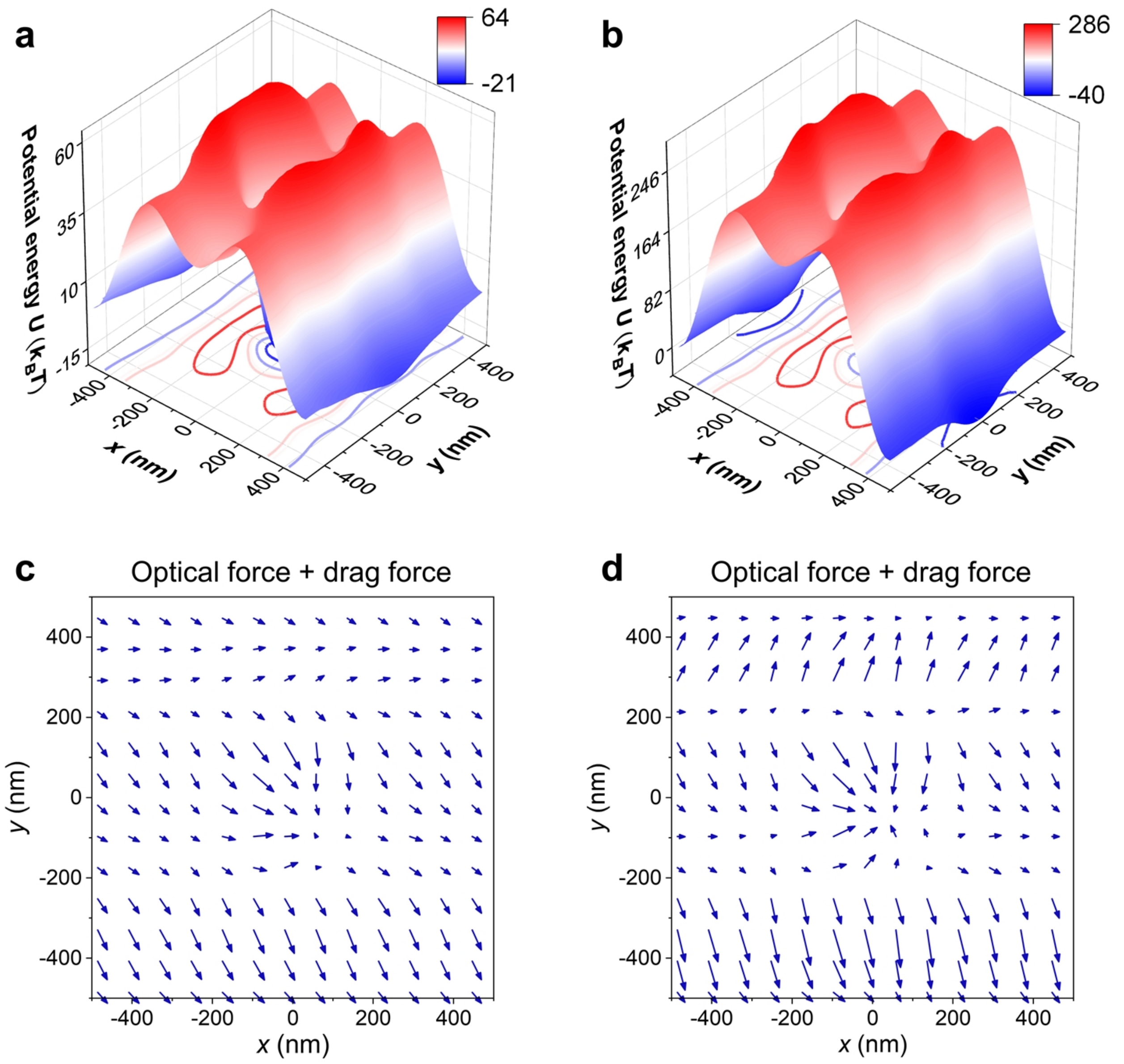

Fig. S6 | Potential wells and total forces for gold nanoparticles in the ED. Potential wells of (**a**) 100-nm and (**b**) 160-nm gold nanoparticles. The potential depth of the 100-nm gold nanoparticle is deeper than the 80-nm gold nanoparticle, thus they can be separated by applying a titled fluid (Fig. 4 in the main text). Total forces (optical force + fluidic drag force) on (**c**) 100-nm and (**d**) 160-nm gold nanoparticles. In (**a**)−(**d**), I = 2.1 mW/μm^2. In (**c**) and (**d**), v = 1500 μm/s.

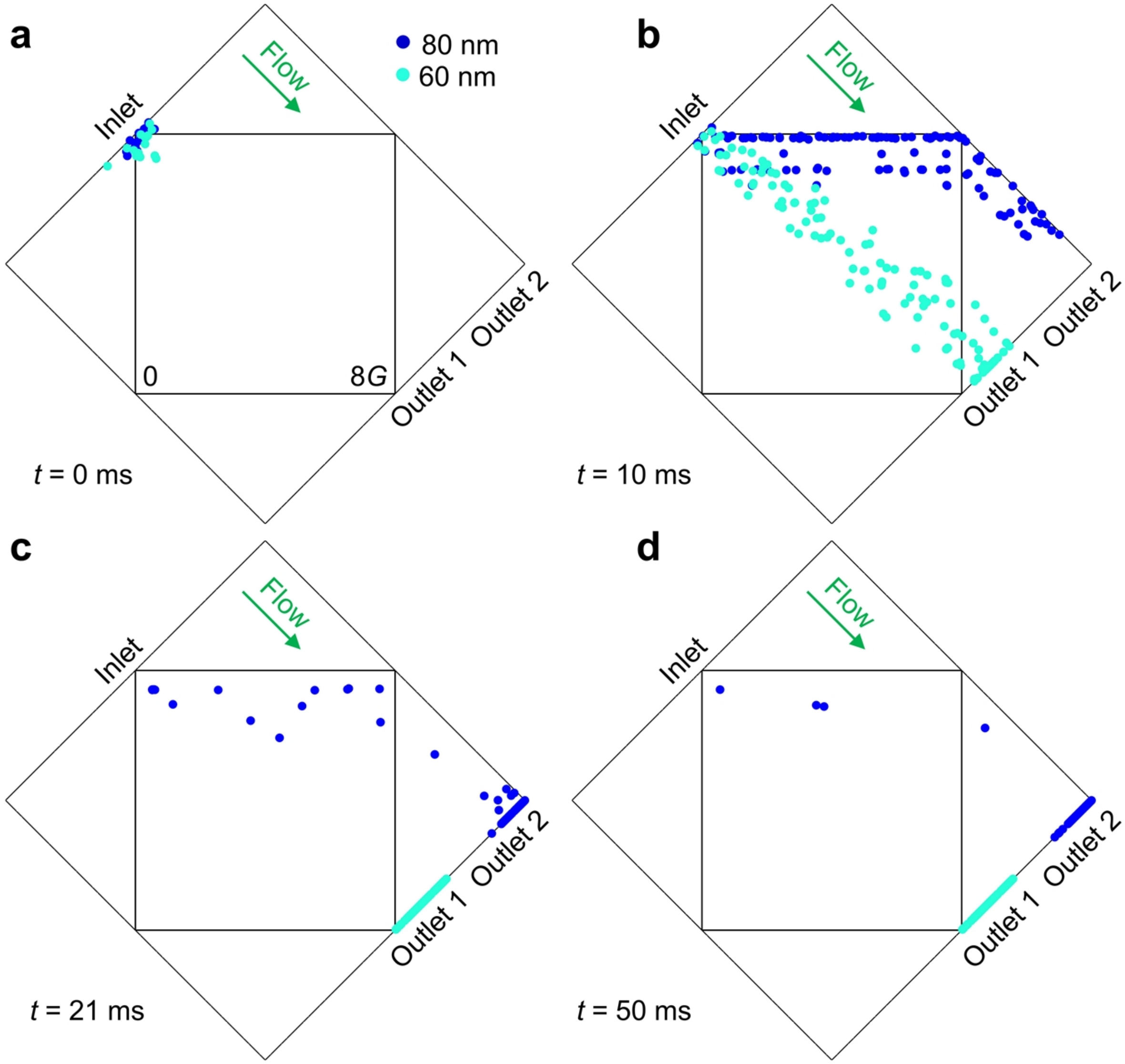

Fig. S7 | Fast sorting and transporting of gold nanoparticles by the photonic slot in the electric dipole mode at the wavelength of 1435.6 nm. Video frames of sorting and transporting gold nanoparticles. 60-nm gold nanoparticles (cyan) move along the fluidic drag force, while 80-nm gold nanoparticles (blue) are confined in photonic slot and transported by the fluidic drag force in the x direction. In (**a**)−(**d**), I = 3.4 mW/μm^2, and v = 1400 μm/s.
